# Navigating dose-effect complexities and challenges in cannabinoid therapy for aging-related neurobiological changes

**DOI:** 10.3389/fphar.2026.1794928

**Published:** 2026-05-28

**Authors:** Ivona-Maria Tudorancea, Leontina-Elena Filipiuc, Diana-Sabina Vieru, Cristina-Elena Dobre, Bogdan-Ionel Tamba

**Affiliations:** 1 Advanced Research and Development Center for Experimental Medicine “Prof. Ostin C. Mungiu” CEMEX, “Grigore T. Popa” University of Medicine and Pharmacy of Iasi, Iasi, Romania; 2 Socola Institute of Psychiatry Iasi, Iasi, Romania; 3 Department of Pharmacology, Clinical Pharmacology and Algesiology, “Grigore T. Popa” University of Medicine and Pharmacy of Iasi, Iasi, Romania

**Keywords:** aging, blood brain barrier, cannabidiol, cannabinoids, neuroinflammation, pharmacokinetic, tetrahydrocannabinol

## Abstract

Growing evidence shows that brain aging is a time-dependent process, with very complex underlying mechanisms at molecular and cellular levels. The endocannabinoid system has emerged as a key regulator of brain homeostasis during aging, interfacing with neuroinflammation, neurogenesis, and blood–brain barrier integrity. However, the neurological impact of exogenous cannabinoids in the aging brain remains incompletely defined and often polarized between neuroprotective and neurodisruptive interpretations. This is a narrative review that critically synthesizes preclinical and clinical evidence on the age-dependent effects of tetrahydrocannabinol (THC) and cannabidiol (CBD), with particular emphasis on dose, route of administration, and pharmacokinetic constraints imposed by aging. Available data indicate that THC exhibits a dual, dose-dependent profile, with low-dose exposure engaging adaptive or potentially neuroprotective mechanisms, whereas moderate to high doses, especially following parenteral administration, are associated with glial activation, neuroinflammatory signaling, and functional impairment. In contrast, CBD demonstrates a seemingly favorable neurological profile in aging models, characterized by anti-neuroinflammatory, antioxidant, and neuroprotective effects, largely independent of CB1R activation. Our findings support the idea that THC and THC predominant therapies may exert a dual effect in the aging brain of experimental models. A similar duality appears to emerge for CBD, particularly with respect to developmental effects, despite insufficient evidence. Collectively, these findings underscore the need for age-adapted, cannabinoid-specific dosing strategies and integrative experimental frameworks to accurately define the therapeutic potential and limitations of cannabinoids in age-associated neurological disorders.

## 1 Introduction

Longevity has long been a topic of intense debate and human curiosity. Until the early 20th century, aging was mainly understood from an evolutionary perspective. Later discoveries, combined with advances in diagnostic techniques and experimental medicine, shaped the main scientific theories of aging. These theories suggest that targeted interventions on specific biological processes and functions that decline with chronological age may attenuate biological aging (Guo et al., 2022). Such an outcome could be beneficial if other major causes of death are controlled, as it might delay mortality associated with pathological conditions. Therefore, current research on longevity aims to identify biological targets that can promote what is known as healthy aging. On the other hand, global demographic projections indicate a marked aging of the world’s population. According to recent United Nations estimates, within the next 3 decades the number of individuals aged 65 years and older will exceed that of the 15–24 age group (United Nations, 2024). This demographic transition underscores the growing need to understand the mechanisms of aging and to develop interventions that promote a healthy long life. Addressing the biological and clinical aspects of aging has thus become a public health and research priority.

Aging manifests overtly in most organ systems through symptomatic processes such as pain, fatigue, and functional impairment. Conditions like hypertension, osteoarticular degeneration, and visceral diseases often produce clinically perceptible signs that accompany aging (Li et al., 2025; Pietri and Stefanadis, 2021). In contrast, neurological aging tends to evolve silently, without early clinical expression at the beginning, which makes it one of the most insidious and potentially dangerous aspects of senescence. Given the rising prevalence of neurodegenerative disorders, including Alzheimer’s disease, Parkinson’s disease, and dementia, advancing research into the neurobiological alterations accompanying aging is imperative (Hou et al., 2019).

The brain constitutes a complex and dynamic microenvironment that, with advancing age, exhibits region-specific changes characterized by neuroinflammation, reduced neurogenesis, impaired neuroplasticity, blood–brain barrier (BBB) dysfunction, oxidative stress and structural remodeling (Blinkouskaya et al., 2021; Knox et al., 2022; Navakkode and Kennedy, 2024). A significant body of evidence suggests that inflammaging (IFG) is recognized as a pivotal convergence point of these mechanisms. IFG occurs when the reparative function of the inflammatory response fails, resulting in a persistent, unresolved inflammatory state (Chung et al., 2019). Moreover, IFG plays a key role in the initiation and progression of neurodegenerative disorders by compromising BBB integrity and inducing neuroinflammatory processes that ultimately drive morphological and functional alterations within the brain (Franceschi et al., 2025; Soraci et al., 2024). At advanced stages, these molecular disturbances lead to clinical manifestations that include behavioral changes. They are also associated with an increased susceptibility to psycho-emotional and memory disorders. Accordingly, neuroinflammation was observed in elderly patients with psychiatric disorders, such as depressive and anxiety-like disorders (Dion-Albert et al., 2022; Leonard and Aricioglu, 2023; Wu and Zhang, 2023).

In this context, increasing attention has been directed toward the endocannabinoid system (ECS) as a potential regulator of neuroinflammatory and degenerative processes associated with IFG. Specific CB1R and CB2R are expressed in the brain, with a notable distribution also within the immune system. Their presence has stimulated extensive *in vitro* and *in vivo* research on endogenous ligands, such as anandamide (AEA) and 2-arachidonoylglycerol (2-AG). Additionally, major exogenous ligands, including CBD -a non-psychoactive compound - and THC - the best-known psychoactive cannabinoid - were intensively investigated for their potential roles in modulating neuroinflammatory and neurodegenerative processes (Haspula and Clark, 2020). Efforts to integrate cannabinoids into clinical research have long been constrained by stringent regulatory frameworks and by the intrinsic challenges associated with achieving consistent product standardization.

Moreover, studies using cannabinoids in aging models have revealed several barriers related to age-dependent physiological changes. These changes directly influence pharmacokinetic processes. Cannabinoids also require careful consideration during administration, as their biopharmaceutical performance (e.g., solubility, stability and bioavailability) depends strongly on the selected formulation and the chosen delivery route. Such variability produces marked differences in pharmacodynamic responses. As a result, studies become highly heterogeneous, which makes it difficult to establish consistent and optimal therapeutic regimens for specific clinical effects. A further consideration in cannabinoid research using aged models is the age-dependent remodeling of the ECS. One of the most robust changes involves the decline in CB1R signaling. Recent evidence demonstrates a progressive reduction in CB1R density and protein expression across cortical regions, indicating a widespread attenuation of endocannabinoid responsiveness in the aging brain (e.g., deep layer VI, medial prefrontal, temporal) (Berrendero et al., 1998; Ginsburg and Hensler, 2022). Moreover, age-associated changes in the activity and expression of endocannabinoid-biosynthetic enzymes have been observed, notably within hippocampal pathways (Piyanova et al., 2015).

Ageing-related studies on cannabinoids remain highly fragmented, with substantial variability in product characteristics (e.g., cannabinoid content/strength and formulation), dosing regimens, ligand profiles, treatment durations, administration route and rodent genetic backgrounds. This heterogeneity restricts mechanistic interpretation and constrains efforts to establish consistent therapeutic principles. This review aims to assemble the existing evidence without attempting to resolve its heterogeneity, but instead to make it more explicit. By outlining differences in dosing regimens, routes of administration, and study designs, it provides a practical reference for future preclinical and clinical research and helps identify gaps in the literature.

## 2 The interplay between neuroinflammation, blood brain barrier dysregulation and neurogenesis in aged brain

Chronological aging is a major risk factor that alters neuronal homeostasis at both morphological and functional levels. With advancing age, peripheral inflammatory processes mediated by specific cytokines exert significant effects on the central nervous system (CNS). BBB serves as a critical interface between the vascular system and the brain parenchyma (Knox et al., 2022). Despite its essential protective role, the BBB remains highly vulnerable to neurotoxins and pathogens. Under physiological conditions, it lacks intercellular clefts, exhibits minimal pinocytotic activity compared with other tissues, and functions as an immunological barrier that restricts the passage of immune cells into the brain. Its integrity depends on tight junction proteins such as occludin and other mlecules including claudins, astrocyte, pericyte, endothelial cells, catenins, transporters and junction adhesion proteins (JAM-1,2,3 and 4) (Knox et al., 2022; Kumar Nelson et al., 2024).

During aging, this regulatory capacity becomes partially impaired. This phenomenon may occur spontaneously, in the absence of an identifiable trigger, or may be promoted by lifelong exposure to chronic inflammatory stimuli or IFG (Erdö et al., 2017). Astrocytic activation is a hallmark of brain aging, resulting in astrogliosis and the release of pro-inflammatory mediators, including interleukin-6 (IL-6) and interleukin-1β (IL-1β) (Knox et al., 2022). Aging also enhances microglial reactivity, leading to a detrimental shift in cellular redox balance. This alteration promotes the excessive production of reactive oxygen species (ROS), pro-inflammatory cytokines such as tumor necrosis factor-α (TNF-α) and nitric oxide (NO). Age-related alterations in pericyte structure and function significantly compromise BBB stability. These mural cells regulate the exchange of molecules between the brain microvasculature and neural tissue, and their gradual dysfunction contributes to increased BBB vulnerability in aging. From a physiological perspective, pericytes have an indirect trophic and metabolic function. They facilitate the exchange of gases and nutrients and contribute to the clearance of residual and toxic compounds in CNS. In the context of aging, increased levels of caspase-3, 1 and 7 promote pericyte apoptosis. The resulting cellular loss compromises BBB integrity and is associated with elevated NO release (Brown et al., 2019; Kumar Nelson et al., 2024). Thus, the BBB becomes permeable to peripheral circulating immune cells (macrophages, lymphocytes, red blood cells) and cytokines. The degradation of BBB facilitates the entry of blood-derived neurotoxic molecules into the brain, including fibrin, thrombin, plasmin, and iron-containing compounds such as hemosiderin. These substances exert direct neurotoxic effects by enhancing oxidative stress, promoting neuronal loss, and driving progressive neurodegeneration. Collectively, these alterations contribute to an accelerated brain aging phenotype (Farrall and Wardlaw, 2009; Levi et al., 2024; Winkler et al., 2014). All these negative consequences manifest at the molecular level through an inflammatory mediated neurodegeneration status. The brain is a complex organ and has several regions. However, the most susceptible to changes in BBB integrity appears to be the hippocampus (Knox et al., 2022; Montagne et al., 2015). Additionally, the role of this region in spatial memory, memory processing and storage, and behavior is well known (Strange et al., 2014).

Unlike peripheral tissues, there are no sensory nociceptors in brain parenchyma. Brain inflammation does not produce the classical signs of redness, heat, pain or swelling. Neuroinflammation expresses itself through functional and behavioral symptoms such as cognitive decline, memory impairment, affective-like disorders and reduced neurogenesis. Neuroinflammation plays a dual role in the aging brain. When brain inflammation is in an acute state and self-limiting, it supports tissue repair and synaptic remodeling. The limit between protective and harmful inflammation is regulated at the molecular level by the activation profile of microglia and astrocytes. Under physiological or acute, self-limiting conditions, microglia contribute to tissue repair, synaptic remodeling, and the maintenance of homeostasis by supporting cell survival, promoting differentiation, and clearing apoptotic cells. Through coordinated interactions with astrocytes, they also regulate immune responses to potentially harmful stimuli, including neurotoxins, injury, and neurodegenerative processes. However, persistent activation of these glial populations shifts the molecular balance toward a pro-inflammatory and neurotoxic phenotype, characterized by sustained release of inflammatory mediators and progressive neuronal loss (Rauf et al., 2022). Although the etiopathogenic mechanisms of neurodegenerative disorders remain incompletely understood, neuroinflammation is recognized as a key contributor to their progression, particularly in Alzheimer’s disease, Parkinson’s disease, and multiple sclerosis (Schain and Kreisl, 2017).

Multiple lines of evidence indicate that intrinsic regulators of adult neurogenesis are epigenetically modulated by extrinsic factors. These factors include physical exercise, psycho-emotional disturbances and lifestyle-related behaviors (Liu et al., 2019; Opendak and Gould, 2015). The molecular mechanisms linking external stimuli to neurogenic regulation are not yet fully elucidated. However, theoretical models and experimental data suggest that molecular changes directing pro- or anti-neurogenic responses remain partially amenable to intervention. Within the hippocampal neurogenic niche, microglia play a central regulatory role. They function as the primary danger-sensing and immune-surveillance cells of the brain. Microglia constitute the first line of immune defense. Through their signaling activity, microglia integrate environmental inputs and shapes the molecular landscape of hippocampal neurogenesis. As previously mentioned, neuroinflammation at the biochemical level is characterized by the activation of microglial and astrocytic cells. This activation is followed by the release of pro-inflammatory cytokines including chemokines, cytokines, and other brain-derived molecules, represent key determinants of microglial functional regulation. Additionally, sustained release of pro-inflammatory mediators has been shown to exert detrimental effects directly on hippocampal neurogenesis. This phenomenon has been predominantly observed within the neurogenic regions of the adult brain. These regions include the subventricular zone and the subgranular zone of the hippocampal dentate gyrus. At this level, neurogenesis is an active process. It is sustained by a pool of immature stem-like cells that represent a key component of the neurogenic niche (Solano Fonseca et al., 2016; Valero et al., 2017). In this context, aging-associated neuroinflammation impairs neuronal differentiation, compromises the survival of hippocampal precursor cells, and alters cerebral endothelial function through the induction of matrix metalloproteinase production (Valero et al., 2017).

Taken together, neuroinflammation, BBB dysfunction, and neurogenesis emerge as tightly interdependent processes. These mechanisms appear to converge on a shared triggering factor, namely, inflammaging. This common pathogenic core positions age-related neurological impairment as a highly relevant subject for investigation. It also provides a coherent framework for examining the documented capacity of cannabinoids to modulate both central and peripheral inflammatory responses (Leonard and Aricioglu, 2023).

## 3 Neuromodulatory effects of cannabinoids

### 3.1 Endocannabinoid signaling in the aging brain

**FIGURE 1:**
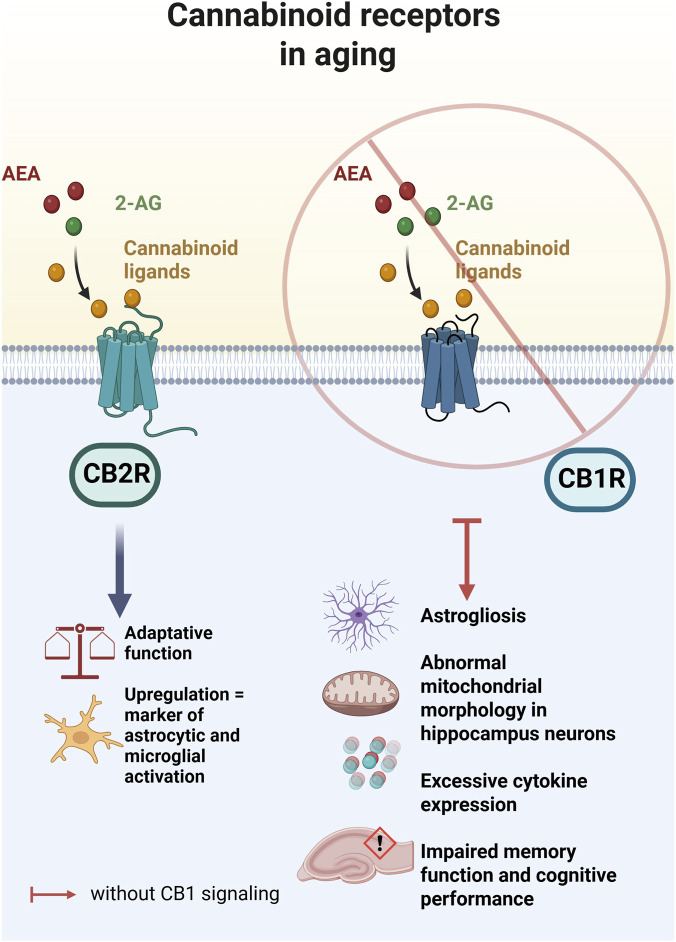
Cannabinoids receptors signaling in aged brain- Illustrative depiction of CB1R and CB2R signaling in preclinical studies of aging, highlighting the relationship between age-associated changes and modulation of the endocannabinoid system. AEA, Anandamide; 2-AG, 2-Arachidonoylglycerol; THC, tetrahydrocannabinol; CBD, cannabidiol; CB1R, Cannabinoid receptor 1; CB2R, Cannabinoid receptor 2. Created in BioRender.

The molecular architecture of the ECS is defined by the presence of the CB1R and CB2R, together with their endogenous ligands, most notably AEA and 2-AG (Haspula and Clark, 2020). Under physiological conditions, CB1R and CB2R exhibit distinct distribution patterns across tissues. However, there is currently no consistent evidence demonstrating age-related differences in their spatial distribution. CB1R are predominantly expressed in central nervous system where they modulate nociceptive processing and are highly represented in regions involved in memory, cognition, and motor control. Specifically, CB1R is expressed in multiple cellular components of the BBB, including astrocytes, pericytes, presynaptic and postsynaptic neuronal compartments, as well as the vascular endothelium (Benyó et al., 2016; Hagan et al., 2022). In contrast, CB2R are primarily localized in peripheral tissues, particularly within immune cells (Minerbi et al., 2019). In contrast to CB1R, CB2R exhibits a distinct central distribution. It is primarily expressed in neurons of the cortex, cerebellum, and brainstem, with variable regional abundance, and shows more prominent localization in perivascular microglia and other components of the neurovascular unit, including endothelial cells, pericytes, and astrocytes (Benyó et al., 2016). Importantly, despite a relatively stable distribution, both receptor density and receptor–effector coupling display age-dependent variation, with additional modulation observed in pathological contexts (Pertwee, 2006). Aging induces heterogeneous alterations in the ECS, with the hippocampus being particularly vulnerable to these changes. Notably, Berrendero et al. described an inverse age-related relationship in the dentate gyrus of the hippocampus, where reduced cannabinoid receptor genetic mediated synthesis coexists with increased receptor binding capacity. This apparent dissociation suggests that aging does not uniformly downregulate cannabinoid signaling but rather induces region-specific adaptations at the receptor level (Berrendero et al., 1998). More recent mechanistic models have proposed that age-related enzymatic alterations leading to reduced 2-AG synthesis constrain endocannabinoid tone and trigger compensatory responses within the system. These responses may include upregulation of receptor binding sites or functional sensitivity increase of CB1R, aimed at preserving signaling efficacy (Piyanova et al., 2015). Numerous studies have shown that alterations in CB1R expression within the brain influence biological processes relevant to aging in rodents. In CB1R knockout mice, an accelerated aging phenotype has been reported. This phenotype is marked at the molecular level by astrogliosis, abnormal mitochondrial morphology in hippocampal neurons, and excessive cytokine expression (Figure 1). In terms of behavior, the phenotype is characterized by reduced cognitive performance and impaired memory function (Kataoka et al., 2020; Marchalant et al., 2007; Marchalant et al., 2009). Age-related changes in CB1R expression and function would be expected to substantially alter the effects of CB1R agonists, particularly in the aged organism. However, available evidence does not consistently support this assumption. Administration of THC, rimonabant, or their combination fails to produce significant behavioral differences between aged and young animals (Ginsburg and Hensler, 2022). These findings indicate that age-dependent remodeling of CB1R populations does not necessarily translate into altered behavioral sensitivity to cannabinoid ligands. Factors such as duration of cannabinoid exposure, dosing strategy, and experimental design are likely to influence the observed responses in aged subjects.

In contrast, CB2R expression in the brain is limited under basal physiological conditions. However, CB2R activation becomes functionally relevant in pathological states, particularly in neurodegeneration. This senescence-favored functional role is closely linked to its predominant localization in microglial cells and its broad distribution within the immune system. Accordingly, CB2R serves an adaptive function, and its upregulation represents a marker of microglial and astrocytic activation during neuroinflammatory processes (Vuic et al., 2022). Current knowledge regarding the involvement of microglial CB2R derives from a broad range of *in vitro* and *in vivo* studies. These investigations include experimental models of Alzheimer’s disease and other neurodegenerative disorders, as well as CB2R knockout models. For example, in an experimental model of Alzheimer’s disease, administration of a CB2R-selective cannabinoid agonist improved cognitive performance. This effect was accompanied by a significant reduction in brain inflammation. The anti-inflammatory response was mediated by decreased expression of pro-inflammatory cytokines, including IL-1β, IL-6, TNF-α, and IFN-γ (Aso et al., 2013). Similarly, in an experimental model of Parkinson’s disease, β-caryophyllene, a cannabinoid like compound, significantly reduced the synthesis of brain pro-inflammatory cytokines, including IL-1β, IL-6, and TNF-α. A modest inhibitory effect was also observed on ciclooxigenase-2 (COX-2) and iNOS expression. These effects were mediated through a CB2R dependent mechanism (Javed et al., 2016). Additionally, in CB2R mice model microglia, inflammatory stimulation fails to induce the typical morphological changes associated with activation. This blunted response reflects an attenuated pro-inflammatory phenotype. It also indicates that CB2R is indispensable for mounting an effective microglia-mediated defense response within the cerebral microenvironment (Reusch et al., 2022).

### 3.2 Cannabinoids as regulators of neural homeostasis during specific age-related model conditions

Research on Cannabis based products (CBP) has expanded substantially in recent years, reflecting growing clinical and scientific interest in their therapeutic potential. In Table 1 we summarize selected experimental evidence supporting the neuroprotective effects of cannabinoids across distinct pathological models. The studies included diverse cannabinoid formulations, doses, and routes of administration. They primarily focus on neuroinflammatory and neurodegenerative disease models. Collectively, these data highlight the capacity of cannabinoids to modulate neuroinflammatory responses, cognitive decline, and disease-associated molecular markers. This comparative overview underscores both the therapeutic potential of cannabinoids and the heterogeneity of their effects, which depend on formulation, treatment regimen, and pathological context.

**TABLE 1:** The neuroprotective effects of cannabinoid-based interventions in age-related neuropathological precinical models.

Treatment	Model	Dose and administration	Treatment period	Main outcomes	Ref.
Combination of 9-THC-enriched botanical extract (67.0% THC) and CBD-enriched botanical extract (62.7% CBD)	12 months-old APP/PS1 mice and 12 months-old WT mice -Trangenic model of advanced Alzheimer’s disease	0.75 mg/kg THC/0.75 mg/kg CBD i.p. injected	Once daily for 5 weeks	Extract reduces memory impairment at advanced stage of AD-like, without effect in WT mice at the same age The combination of extracts suppresses long-term plasticity mechanisms that underlie learning and memory	Aso et al. (2016)
THC in vehicle (the actual form of THC is not mentioned)	14–17-month-old-Trangenic model of Alzheimer’s disease (APP/PS1 transgenic mice)	0.2 and 0.02 mg/kg i.p. injected	Once daily for 3 months	Increased the expression of Aβ monomers and phospho-GSK-3β (Ser9) in the THC-treated brain tissues	Wang et al. (2022)
CBD extract oil (81	12-month-old SAMP8 mice	3 and 30 mg/kg Oral gavage administration	Once daily for 8 weeks	CBD highlighted an anxiolytic effect in the elevated plus maze test CBD improved memory impairment in NOR and T-maze	Goodland et al. (2025)
THC + CBD	C57BL/6 female mice aged 6–8-week-old -Experimental model of autoimmune encephalomyelitis	10 mg/kg +10 mg/kg i.p administration	Once daily for 1 week	Reduction in IL-17A, IFN-γ, TNF-α, IL-6, and IL-1β production from cell cultures from brain tissue Increased levels of IL-10 and TGF-β	Al-Ghezi et al. (2019)
CBD with vehicle (2% Tween-80% and 98% saline)	Adult male Wistar rats-Experimental model of Alzheimer disease	0.1 mg/kg and 1 mg/kg i.p administration	Once daily for 2 weeks	Reduction in TNF-α, NFkB1, IL-1β pro-inflammatory markers CBD prevents cognitive and social impairments Reduced hippocampal Aβ accumulation, p-tau, TREM2, and ApoEɛ4	Toledano and Akirav (2025)

i.p, intraperitoneally; AD, Alzheimer’s disease; THC, tetrahydrocannabinol; CBD, cannabidiol; GSK-3β, -Glycogen synthase kinase-3 beta; in IL-17A-interleukine 17A; IFN-γ, interferon-gamma; IL-6, interleukine-6; IL-10, interleukine-10; TNF-α, tumor necrosis factor α; IL-1β, interleukin 1β; NFkB1, Nuclear Factor Kappa B Subunit 1; NOR, Novel object recognition test; Ref., references; SAMP8 mice, Senescence-Accelerated Mouse Prone 8; TGF- β, Transforming growth factor-beta; TREM2, Triggering Receptor Expressed on Myeloid cells 2; WT, wild type.

### 3.3 Cannabidiol as a modulator of inflammaging: systemic and neuroprotective outcomes

Clinical investigation of CBD has expanded substantially in recent years. In older populations, reported neurobiological benefits include the improvement of behavioral disturbances, particularly agitation, aggression, apathy, and sleep disorders. In a randomized, placebo-controlled clinical trial, a CBD-enriched cannabinoid phytocomplex improved behavioral symptoms in patients over 60 years of age with various forms of dementia. The intervention demonstrated a favorable safety and efficacy profile, with significant reductions in agitation, aggression, and sleep-related disturbances (Hermush et al., 2022). Consistent with these findings, a pilot clinical study evaluating CBD in elderly patients aged 65–90 years with behavioral and psychological symptoms of dementia, including Alzheimer’s disease, reported good tolerability following administration of 200 mg oral capsules. Notably, a reduction in agitation emerged as a clinically relevant outcome warranting further investigation (Velayudhan et al., 2024). Beyond neuropsychiatric manifestations, modulation of the endocannabinoid system has also been proposed as a therapeutic strategy for chronic neuropathic pain in older adults. In this context, CBD has attracted particular interest, representing a promising direction for future clinical research in geriatric populations (Hakami and Alshehri, 2025).

Experimental models have demonstrated that CBD modulates key processes implicated in aging. Several evidence indicates that CBD modulates the systemic consequences of IFG during biological aging. For example, in aged Long Evans rats (15 months), daily administration of CBD at a dose of 10 mg/kg for 10 weeks improved splenic leukocyte function and enhanced systemic antioxidant defense. These effects were primarily driven by modulation of glutathione-dependent redox pathways, as reflected by a reduction in the GSSG/GSH ratio (oxidized glutathione/reduced glutathione) and decreased lipid peroxidation in both circulating blood cells and splenic tissue. Notably, the antioxidant benefits of CBD in the aging organism appear to be tissue-dependent, with more pronounced effects observed in the liver compared with the lung and thymus, particularly with respect to the regulation of glutathione redox homeostasis (De la Fuente et al., 2024). Furthermore, administration of CBD at this dose effectively counteracted aging-associated increases in pro-inflammatory mediators in both hepatic and pulmonary tissues, including TNF-α, IL-1β, and Nuclear Factor kappa-light-chain-enhancer of activated B cells (NF-κB). This anti-inflammatory effect was accompanied by attenuation of apoptosis-related signaling, as evidenced by reduced caspase-1 levels in the liver and lungs of 15-month-old rats (Rancan et al., 2023). These results indicate that by attenuating chronic low-grade inflammation and associated oxidative stress, CBD contributes to the regulation of age-related inflammatory pathways across multiple organ systems. This multimodal activity highlights the potential of CBD to influence key systemic processes underlying aging.

**FIGURE 2:**
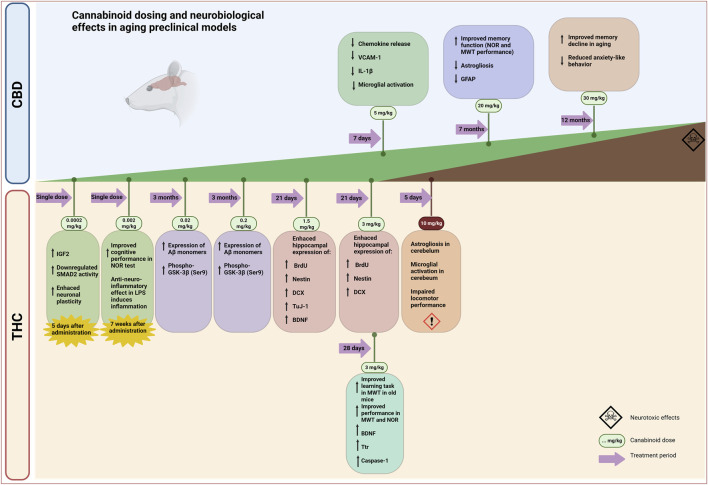
Cannabinoid dosing and neurobiological effects in aging preclinical models- Schematic overview of selected preclinical findings derived from rodent studies, illustrating the effects of parenterally administered CBD and THC in experimental models relevant to aging. BDNF, Brain-derived neurotrophic factor; BrdU, Bromodeoxyuridine; CBD, Cannabidiol; COX-2, Cyclooxygenase-2; DCX, Doublecortin; GFAP, Glial fibrillary acidic protein; GSK-3β, Glycogen synthase kinase 3β; IGF-2, Insulin-like growth factor-2; IL-1β, Interleukin-1β; LPS, Lipopolysaccharide; THC, Tetrahydrocannabinol; Ttr, Transthyretin; TuJ-1, Neuron-specific class III beta-tubulin; VCAM-1, Vascular cell adhesion molecule-1. Created in BioRender.

Based on the available evidence, there are currently no *in vivo* studies demonstrating neurotoxic effects associated with chronic CBD administration in preclinical models of aging. On the contrary, CBD consistently exhibits neuroprotective properties mediated through multiple, partially elucidated mechanisms. Supporting these observations, Mirza Agha et al. reported that chronic CBD treatment (20 mg/kg body weight for 7 months) also modulates the cerebral inflammatory response in aging. In their study, daily oral administration of CBD for 7 months in C57BL/6 mice, aged 14 months at study onset, led to significant improvements in performance in the novel object recognition (NOR) and Morris Water maze (MWT) tests. These behavioral effects were associated with reduced neuroinflammation and decreased expression of the astrocytic activation marker glial fibrillary acidic protein (GFAP) in the hippocampus, suggesting an improvement in memory function mediated by attenuation of glial reactivity (Mirza Agha et al., 2025). Consistent findings were observed in a model of accelerated senescence, where chronic administration of CBD at 30 mg/kg/day in 12-month-old SAMP8 mice improved both memory performance and anxiety-related behavior (Figure 2). Biochemical analyses in this model revealed a reduction in oxidative stress markers, indicating that the cognitive benefits of CBD are closely linked to decreased oxidative burden. Notably, CBD significantly lowered levels of 4-hydroxynonenal, a byproduct of lipid peroxidation, further supporting its role in mitigating oxidative stress associated with the aging phenotype (Goodland et al., 2025). From a pharmacological perspective, the molecular mechanisms underlying the effects of CBD are not yet fully elucidated. However, current evidence indicates that these effects are not mediated by direct activation of CB1R, but rather by indirect modulation of endocannabinoid signaling. CBD inhibits the enzymatic hydrolysis of AEA at the synaptic level, thereby enhancing endocannabinoid tone and promoting CB2R-dependent anti-neuroinflammatory, immunomodulatory, and antioxidant effects in the brain (Singh and Neary, 2020).

Taken together, these preclinical findings provide compelling evidence for the neuroprotective and anti-neuroinflammatory properties of CBD. However, its impact on BBB function remains insufficiently characterized in the literature. Notably, data derived from *in vivo* aging models addressing direct effects of CBD on BBB integrity are largely lacking. Indirect evidence arises from a murine model of multiple sclerosis, in which sub-chronic administration of CBD (5 mg/kg/day for 7 days) significantly reduced the production of chemokines (CCL1,2) that act as key chemo-attractants for inflammatory cells within the CNS. Additionally, CBD treatment reduces the expression of vascular cell adhesion molecule-1 (VCAM-1), thereby contributing to the strengthening of the BBB. In this context, CBD also limited lymphocyte recruitment by immunomodulating astrocytic and microglial inflammatory responses, thereby attenuating immune-mediated cerebral processes known to compromise BBB integrity (Mecha et al., 2013). Complementary *in vitro* studies using co-cultures of human brain microvascular endothelial cells and human astrocytes exposed to oxygen–glucose deprivation demonstrated that CBD at 10 μM prevented the oxygen–glucose deprivation -induced increase in barrier permeability. This protective effect was mediated through activation of PPARγ and, in part, 5-HT1A receptors, and was accompanied by reduced expression of vascular cell adhesion molecule-1 and IL-6 in endothelial cells (Hind et al., 2016). Collectively, these findings suggest that CBD may exert barrier-stabilizing effects by limiting inflammatory and immune mechanisms that undermine BBB function, although direct validation in aging-specific *in vivo* models remains an important gap to be addressed.

On the other hand, available preclinical evidence indicates that the effects of CBD on neurogenesis are dose dependent and follow an inverted U-shaped response curve. In experimental models, CBD has been shown to enhance neuronal proliferation and differentiation at doses ranging between 3 mg/kg and 30 mg/kg body weight in a linear dose–response manner. Outside this window, both lower and higher doses are associated with a loss or reversal of these proneurogenic effects, as presented in a recent review (Luján and Valverde, 2020). Although the underlying mechanisms remain incompletely understood, several converging pathways have been proposed. This dose–response profile supports the existence of a direct mechanism by which CBD influences neurogenesis. Proposed pathways include modulation of the ECS, particularly activation of CB2R, as well as inhibition of FAAH, leading to increased levels of AEA. Additionally, activation of PPARγ receptors may contribute to the neuroprotective and proneurogenic actions of CBD. However, the functional relevance of PPARγ signaling alone does not fully account for the loss of efficacy observed at doses below 3 mg/kg or above 30 mg/kg (Valeri and Mazzon, 2021). In parallel, an indirect mechanism is likely to contribute. CBD may support neurogenesis by creating a favorable hippocampal microenvironment for neural progenitor cell proliferation and survival. This effect may be mediated by the attenuation of oxidative stress and neuroinflammation, two processes known to impair adult hippocampal neurogenesis (Russo et al., 2011). By reducing these inhibitory factors, CBD may indirectly enhance the proliferation and differentiation of hippocampal neural stem cells, thereby complementing its direct molecular actions. Importantly, most of the evidence supporting the proneurogenic effects of CBD derives from longitudinal studies performed in young adult rodent models. Consequently, the extent to which these findings can be extrapolated to aged organisms remains insufficiently explored. In this context, a more systematic evaluation of potential age-dependent responses to CBD may represent an important avenue for future research, particularly with respect to hippocampal neurogenesis in aging models.

Interestingly, in zebrafish models, early-life exposure to CBD appears to induce pronounced long-term alterations in behavior with cognitive and gene expression impairments consequences, in some cases exceeding those observed with THC (Achenbach et al., 2018; Carty et al., 2019). Additionally, developmental CBD exposure has been shown to affect metabolic and reproductive parameters, despite concomitant beneficial effects such as increased survival and reduced inflammatory markers (Pandelides et al., 2020). These findings suggest that, although CBD is widely regarded as a neuroprotective compound with a favorable safety profile, its biological effects are more complex and may include previously underrecognized long-term consequences. In this context, emerging preclinical evidence indicates that CBD may influence age-related processes, warranting a more cautious and nuanced interpretation of its neuroprotective potential.

### 3.4 Dual effect of THC in age-related neurological conditions

A substantial proportion of the evidence supporting negative neuromodulatory effects associated with cannabinoid exposure derives from clinical and observational studies conducted in human users rather than from controlled preclinical experiments. THC acts as a partial agonist at CB1R and CB2R within the endocannabinoid system, with predominant central expression, thereby modulating neurotransmission and accounting for its psychotropic effects. For that reason, the reported adverse outcomes predominantly affect the CNS and are characterized by memory, attention, and executive function impairments, particularly in the context of long-term or heavy *Cannabis* use (Nain et al., 2025). Adverse effects associated with CBP use may be attributable, at least in part, to the administration of THC through smoking, a route that has been consistently associated with an increased risk of *Cannabis*-related mental health disorders (de Bode et al., 2025). Nevertheless, the route of administration is frequently not controlled in clinical studies and meta-analyses, a limitation explicitly acknowledged by several authors. For example, in a meta-analysis examining non-acute effects of *Cannabis* use, the route of administration was not considered, as the included studies relied on naturalistic patterns of consumption rather than controlled pharmacological exposure (Yanes et al., 2018). While the findings suggest that CBP use may be associated with altered neural correlates of cognitive control, based on neuroimaging data, the route of administration and other key pharmacokinetic variables were not addressed. Accordingly, these conclusions should be interpreted with careful consideration of the pharmacological and neurobiological implications of *Cannabis* administration routes. Additionally, the timing of exposure, particularly during critical periods of brain development, appears to be a major determinant of vulnerability. Studies examining older adults between 55 and 75 years of age indicate that negative neurological outcomes are more pronounced in individuals with a history of chronic *Cannabis* consumption initiated during adolescence or early adulthood, specifically before the age of 20 years old. In this population, prior early-life exposure has been associated with persistent structural alterations, including reduced gray matter volume in hippocampal regions implicated in memory processing, suggesting long-lasting consequences that may manifest or be exacerbated later in life (Burggren et al., 2018). Additionally, regular *Cannabis* exposure has been associated with region-specific alterations in neural connectivity, particularly in brain regions with high CB1R density, including the medial temporal cortex, the temporo-parietal-occipital junction, and the hippocampus. In the study reporting these findings, the term “*Cannabis* users” was not defined with respect to the route of administration (Soleimani et al., 2023). As a result, the observed effects likely reflect exposure patterns that differ from those used in experimental cannabinoid research, where administration is tightly controlled. These differences limit direct comparability with preclinical models, where intraperitoneal, subcutaneous, or non-thermally processed oral administration for cannabinoids is typically used. Building on this discrepancy, such preconceptions may contribute to the development of a persistent stigma surrounding the neuromodulatory capacity of THC, whereby its effects are predominantly interpreted as neurodisruptive. Consequently, a dominant interpretative framework is reinforced, which remains difficult to revise despite consistent preclinical evidence demonstrating dose-dependent and, under controlled conditions, potentially neuroprotective effects of THC, even when administration approaches are adapted toward clinically relevant conditions. To improve translational validity, future experimental studies should increasingly incorporate administration routes and pharmacokinetic conditions that more closely reflect clinical practice, thereby enabling a more accurate integration of preclinical and clinical findings.

Preclinical investigations of THC provide a more nuanced and mechanistically informative perspective on its neurological effects. Experimental studies in animal models consistently indicate a dual profile of THC action, characterized by either neuroprotective or reversible neurotoxic outcomes depending on some important details including dose, duration of exposure and developmental stage. However, the heterogeneity of results from preclinical studies on the neurological effects of THC is largely due to the uneven distribution of CB1R in the brain (Herkenham et al., 1990). In this pharmacological context, the time of exposure to THC will lead to region-specific changes as well (Mohammadpanah et al., 2023). Variability in the physiological response to THC has been conceptualized as the biphasic effect of cannabinoids, a phenomenon first proposed in the second half of the 20th century (Turkanis and Karler, 1981). Early observations indicated that low and high doses of THC elicit opposing behavioral outcomes. These dose-dependent effects were subsequently confirmed across multiple functional domains, including pain processing, anxiety, and cognition (Lichenstein, 2022; Zhang-James et al., 2023). Similar biphasic responses have been reported for other CB1R agonists, such as WIN55,212-2. In this case, low doses (0.5 mg/kg, i.p.) increase hippocampal acetylcholine efflux, whereas higher doses (5 mg/kg, i.p.) produce opposite effects (Tzavara et al., 2003). Collectively, these findings establish the biphasic response as a fundamental feature of cannabinoid pharmacology. This property underlies the contemporary interpretation of cannabinoids as agents with either neuroprotective or neurodysfunctional effects, depending on dose, context, and the stage of brain development. Consistent with this framework, parenteral administration of THC at 10 mg/kg for 5 consecutive days in a murine model induced astrogliosis and microglial activation in the cerebellum. These changes were accompanied by impaired locomotor performance. At this dose and in this brain region, THC therefore does not appear to exert beneficial effects (Mohammadpanah et al., 2023). In contrast, the detrimental effects observed at higher THC doses appear to be attenuated by co-administration with CBD. Specifically, a combination of 10 mg/kg body weight THC and 10 mg/kg CBD exerted neuroprotective effects *in vitro*. This protection was mediated by an anti-neuroinflammatory response, characterized by reduced levels of IL-17A, IFN-γ, TNF-α, IL-6, and IL-1β, together with increased production of IL-10 and TGF-β (Al-Ghezi et al., 2019).

At very low doses, THC has been reported to exert beneficial effects on neurogenesis, particularly within hippocampal regions. Chronic administration of 1.5 mg/kg and 3 mg/kg THC body weight was associated with enhanced hippocampal expression of BrdU (involved in division of neuronal progenitor cell), BDNF, nestin, DCX (doublecortin), and TuJ-1, markers involved in neuronal progenitor proliferation, differentiation, and survival, suggesting modulation of neuronal regeneration processes (Suliman et al., 2017). Moreover, even lower doses of THC (0.2 and 0.02 mg/kg body weight), administered for 3 months in a transgenic mouse model of Alzheimer’s disease, increased the expression of Aβ monomers and phospho-GSK-3β (Ser9) in brain tissue. Conversely, beneficial effects of THC have been reported following administration as a standardized phytocomplex containing 15% THC and THC precursor THCA-A, produced under EU-GMP certification. In this context, our group previously demonstrated that administration of this phytocomplex at doses of 6.25 mg/kg and 25 mg/kg body weight in aged rats significantly reduced neuroinflammatory markers, including TNF-α, COX-2, and IL-1β, within the dentate gyrus of the hippocampus. Additionally, the phytocomplex appeared to modulate adult hippocampal neurogenesis in the subgranular zone, as indicated by changes in caspase expression (Tudorancea et al., 2025).

Interestingly, microdosing in preclinical THC treatment appears to be a paradigm shift in the pharmacology of these compounds. Microdosing of THC was reported to be 3 to 4 orders of magnitude lower than doses producing conventional cannabinoid effects in rodents. The administration of an ultra-low dose of THC (0.0002 mg/kg body weight) induced a marked increase in insulin-like growth factor 2 (IGF2), a peptide known to decline with age and to exert neuroprotective effects, particularly in hippocampus-dependent memory and cognitive processes. This increase was detected 5 days after THC administration. In parallel, THC modulated the SMAD2 signaling pathway by downregulating its activity, a mechanism associated with enhanced axonal growth and neuronal plasticity (Gradari et al., 2021; Shapira et al., 2023) (Figure 2). Collectively, these findings suggest that THC microdosing may positively influence neuroplasticity in aged animals by promoting neuronal proliferation and maturation within the dentate gyrus of the hippocampus. Upon a tenfold increase in dose, Tselnicker et al. demonstrated that administration of 0.001 mg/kg body weight THC resulted in a sustained impairment of cognitive performance in mice, as evaluated using the Morris Water Maze, with effects persisting for at least 3 weeks (Tselnicker et al., 2007). Notably, a further increase in dose to 0.002 mg/kg body weight was associated with a restoration of neuroprotective effects, underscoring the dual, dose-dependent nature of THC action. Consistent with this observation, a single administration of 0.002 mg/kg THC conferred sustained anti-neuroinflammatory effects in an experimentally induced inflammation model, with protection persisting for up to 7 weeks. This intervention was also associated with significant improvements in cognitive performance. Evidence indicates that, in this context, the anti-neuroinflammatory action of THC is mediated predominantly via CB1R activation (Fishbein-Kaminietsky et al., 2014). Interestingly, the same microdose of THC provided protection against neuronal damage induced by diverse neurotoxic insults, including the epileptogenic agent pentylenetetrazol, the anesthetic pentobarbital, carbon monoxide–induced hypoxia, and the psychostimulant 3,4-methylenedioxymethamphetamine (MDMA). Importantly, the magnitude of neuroprotection depended on the timing of THC administration, with maximal effects observed when THC was administered 24 or 48 h prior to the neurotoxic insults. These observations have led to the proposal of a preconditioning-like mechanism attributed to THC. Specifically, exposure to ultra-low doses may act as a mildly noxious stimulus, priming endogenous neuroprotective pathways and thereby enhancing resilience to subsequent neuronal injury (Assaf et al., 2011; Fishbein et al., 2012). Accordingly, Sarne et al. investigated whether age-related neurological alterations could be modulated by ultra-low doses of THC, and their findings supported this hypothesis. THC has been shown to ameliorate age-related cognitive deficits in old mice, while inducing subtle but measurable adverse effects on young animals. In other words, it should be noted that the neuroprotective effect of THC microdoses may be age dependent (Sarne et al., 2018). Collectively, these examples indicate that the neuroprotective effects of THC microdosing have been achieved primarily following single-dose administration. However, it remains to be determined whether such effects are sustained under chronic treatment with ultra-low THC doses, particularly in the context of aging animal models. These findings support a potential anti-neurodegenerative and neuroprotective role of low-dose and ultra-low dose of THC in murine models (Wang et al., 2022). THC also modulates neuroplasticity in the rodent brain. In adolescent rats subjected to behavioral training, THC reduced the transcription of neuroplasticity-related markers. These effects were clearly dependent on both dose and age (Steel et al., 2014).

In conclusion, available preclinical evidence suggests that the neuromodulatory actions of THC are characterized by a dual, largely dose-dependent profile. From a pharmacological and neuropathogenic perspective, THC appears to differ substantially from classical recreational drugs, indicating that the use of the term neurotoxicity may be of limited conceptual accuracy in this context. Rather, the notion of neuromodulatory dysfunction may better capture the nature of THC-induced central effects. Importantly, the undesirable neurological outcomes associated with THC exposure should not be directly equated with those induced by highly addictive illicit substances such as cocaine or methamphetamine. These agents are known to produce pronounced neurotoxic effects through well-characterized mechanisms, including oxidative stress, excitotoxicity, neuroinflammation, mitochondrial dysfunction, cerebrovascular alterations, and subsequent neuronal loss with overt structural damage (Wang et al., 2025). In contrast, preclinical data reveals a complex, dose- and context-dependent profile of THC action, strongly influenced by regional CB1R distribution, developmental stage, and exposure parameters. The biphasic nature of cannabinoid pharmacology emerges as a central framework, reconciling neurodysfunctional effects at higher doses with neuroprotective or pro-neurogenic outcomes at low or ultra-low doses, especially within hippocampal circuits. Moreover, the modulation of THC effects by CBD further emphasizes the importance of cannabinoid interactions in shaping neurobiological outcomes. Collectively, these findings argue against a unidimensional interpretation of THC neurotoxicity and support a nuanced, dose-sensitive perspective that is essential for translational relevance, risk assessment, and the rational design of cannabinoid-based therapeutic strategies.

## 4 Cannabinoids - pharmacological challenges in translating preclinical findings to clinical practice

### 4.1 Barriers to the standardization of Cannabis-based products

Cannabinoids are naturally derived molecules produced by *Cannabis* species, which encompass more than 150 identified cannabinoids together with a range of other phytochemicals, including terpenes, flavonoids, and polyphenols. These constituents function as cannabimimetic ligands and can interact to modulate biological activity in a manner that exceeds the effects of the individual compounds. This coordinated activity is commonly described as the “entourage effect” (LaVigne et al., 2021). This term encompasses the phenomenon where specific plant metabolites can modulate the pharmacological effects of individual bioactive substances, despite lacking inherent pharmacological activity themselves (Cogan, 2020). Therefore, starting from 1999, Mechoulam and Ben-Shabat proposed the concept that combinations of bioactive substances derived from plants may exhibit greater pharmacological efficacy compared to individual components. Consequently, this interaction suggests effects that may be unpredictable without rigorous experimental studies that are carefully designed and controlled (Al-Khazaleh et al., 2024; Cogan, 2020). The phytochemical composition of *Cannabis* species varies substantially across strains, influenced by differences in cannabinoid proportions as well as by cultivation, storage, and processing conditions. Beyond cannabinoids, Cannabis contains a diverse array of terpenes, flavonoids, and other minor constituents that may further contribute to this modulatory framework. Several studies indicates that terpenes may modulate pharmacodynamic responses by interacting with neurotransmitter systems, influencing receptor signaling, and potentially influence the BBB permeability, which in turn may impact the bioavailability and activity of major cannabinoids (Al-Khazaleh et al., 2024; Baron, 2018). Moreover, variability in extraction methods and formulation approaches may significantly impact the relative abundance and stability of these compounds. This complexity underscores the difficulty in attributing observed biological effects to single molecules and highlights the need for standardized formulations and well-controlled studies to better delineate the contribution of individual components within the broader phytochemical matrix (Al-Khazaleh et al., 2024).

In the original plant, cannabinoids are present predominantly in their acidic forms, for example, tetrahydrocannabinolic acid (THCA), cannabidiolic acid (CBDA), and cannabigerolic acid (CBGA) (Filer, 2022). Although these acidic precursors were long regarded as pharmacologically inactive, recent studies have demonstrated that they exert specific biological effects. Notably, the non-psychotropic precursor of THC (THCA) has shown anti-inflammatory, neuroprotective, and antiemetic activity in preclinical models (Anderson et al., 2021; Moreno-Sanz, 2016; Nadal et al., 2017; Palomares et al., 2020). A recurring source of confusion in preclinical and clinical studies involving natural cannabinoids concerns the nomenclature of THC-related acidic species. The acidic form present in the plant is a biochemical precursor of THC that, upon decarboxylation, yields the neutral compound. This precursor is often conflated with THC-COOH, an oxidative metabolite produced *in vivo* through hepatic biotransformation. The similarity in abbreviated chemical designations contributes substantially to this confusion. Although the more accurate notation for the plant-derived precursor is “THCA-A,” the simplified abbreviation THCA is frequently used, making it easy to mistake it for THC-COOH, whose abbreviation is chemically correct and widely accepted. The consequences of these inconsistencies are evident in literature. Rosenthaler and colleagues refer to the administered compound as THCA, while the structure they are presenting in the article corresponds to THCA-A (Rosenthaler et al., 2014). Likewise, Nadal et al. use THCA when describing the precursor of THC, although their data indicate they are examining THCA-A (Nadal et al., 2017). In reality, THCA and THCA-A are commonly used to denote the same compound (the carboxylated precursor of THC); however, alternating between the two abbreviations within and across studies can be needlessly confusing. On the other hand, some authors refer to the metabolite 11-hydroxy-Δ^9^-tetrahydrocannabinol by the name THCA, which further amplifies this confusion (Coulter et al., 2008).

Another source of ambiguity concerns the reported THC content of cannabis-based products (CBPs) when calculated using the Ph. Eur. approach, which defines total THC as the sum of THC and its acidic precursor THCA-A. This convention can create the misleading impression that a product contains substantially more psychoactive THC than it actually does, particularly when the cannabinoid profile is dominated by THCA-A, a non-psychoactive precursor that may be present in much higher proportions. This approach hides the variability of decarboxylation, a process influenced by temperature, light, storage, and processing (Filer, 2022). Special attention should be given to the fact that THCA does not undergo decarboxylation *in vivo* but only in response to external physical factors such as those noted above (Moreno-Sanz, 2016). As a result, the proportion between active cannabinoids and their acidic forms may shift unpredictably. Because these species differ in stability, bioavailability, and pharmacological effects, the true dose of the active compound becomes difficult to determine. In clinical research, this uncertainty reduces batch-to-batch comparability and complicates the interpretation of dose–response relationships. It can ultimately weaken the strength and reliability of conclusions about the safety and efficacy of natural Cannabis preparations. This issue is illustrated by our pharmacokinetic observations following oral administration of an EU-GMP–certified *Cannabis sativa L.* phytocomplex. According to the Ph. Eur. monograph, the tested product qualifies as high-potency preparation, containing 15% total THC. However, this value likely reflects a predominance of THCA-A within the declared content. Consistent with this interpretation, serum THC remained undetectable after oral doses of 5 mg/kg and 50 mg/kg body weight, while THCA-A reached high circulating concentrations. Detectable systemic levels of THC only emerged after administration of substantially higher doses, exceeding 300 mg/kg body weight (Filipiuc et al., 2023). These findings indicate a marked dissociation between nominal product potency, as defined by the Ph. Eur. methodology, and actual systemic exposure to psychoactive THC following oral administration. Together, these observations highlight a critical limitation of the current Ph. Eur. dosing framework, whereby products classified as high potency based on total THC content may not confer corresponding psychoactive exposure under typical oral dosing conditions. A clearer regulatory distinction between THC and THCA-A would improve dose definition, enable more accurate risk assessment, and support a more rational interpretation of pharmacokinetic and safety data in both research and regulatory settings.

Therefore, the overlapping and imprecise use of abbreviations for THC acidic derivatives, exacerbated by regulatory conventions, introduces unnecessary ambiguity and increases the likelihood of misinterpretation. Adoption of a clear and uniform nomenclature, or at least a consensus that THCA refers exclusively to THCA-A, is essential to improve accuracy, reproducibility, and comparability across studies.

### 4.2 Pharmacokinetic and pharmacodynamic challenges in aging context

Over the past decades, cannabinoid research has advanced rapidly, with particular focus on characterizing the ECS (Hoch et al., 2024). Accumulating evidence indicates that ECS dysregulation contributes to neurological, psychological, and physiological impairments. Conversely, administration of specific cannabinoids, under defined dosing and delivery conditions, has been shown to improve neuromotor function, cognitive performance, and neuroinflammatory outcomes. The relevance of cannabinoids in the study of neuropathology is widely recognized. However, their investigation and therapeutic application are constrained by several pharmacokinetic and pharmacological limitations.

Cannabinoids are highly lipophilic compounds with variable bioavailability, influenced by species, formulation, route of administration, dose, and food intake (Kaszewska et al., 2025; Millar et al., 2018). For instance, the oral bioavailability of THC is approximately 6%, whereas inhalational administration increases bioavailability by nearly fivefold (Naef et al., 2004). Pharmacokinetic data for THC and CBD are largely derived from preclinical studies. Clinical investigations focusing on this pharmacological stage in vulnerable populations, particularly individuals older than 65 years, remain scarce. The available clinical data indicates a clear dose-dependent pattern of adverse effects. When THC was administered orally in tablet form at a dose of 6.5 mg, patients reported drowsiness and dry mouth more frequently than those receiving 3 mg or 5 mg THC, or placebo (Ahmed et al., 2014). Moreover, in individuals with dementia, daily doses of 0.75 mg and 1.5 mg THC were well tolerated (Ahmed et al., 2015). Consistent with these findings, additional studies reported that a daily dose of 2.5 mg THC (dronabinol) was safe and well tolerated in elderly patients with dementia (Beal et al., 1997; Walther et al., 2006).

Preclinical models offer valuable insights for dissecting the variability associated with cannabinoid dosing strategies in the context of aging, but these are not without limitations. In aged animal models, differences related to dose, dosing regimen, and route of administration can be systematically controlled and directly compared, allowing subtle pharmacological effects to be more readily detected than in clinical settings. This experimental flexibility facilitates the identification of dose-dependent and regiment-specific outcomes that are particularly relevant in the aged nervous system. To capture this variability, we synthesized the available evidence in a summary table, compiling preclinical studies that we considered representative of this field. These studies consistently report beneficial neurological effects of THC and CBD in aging, supporting the relevance of cannabinoid-based interventions in age-related neurobiological decline. As summarized in Table 2, preclinical studies in aged animal models revealed a potential relationship between dosing strategy, route of administration, and neurological outcomes. Low-dose THC administered chronically via parenteral routes (e.g., 1–3 mg/kg/day delivered subcutaneously through osmotic minipumps) consistently improved learning, memory performance, and synaptic plasticity markers in aged mice (Kataoka et al., 2020; Komorowska-Müller et al., 2023; Nidadavolu et al., 2021).

**TABLE 2:** Experimental evidence supporting neuroprotection by Cannabis-based products in animal models of natural or chemically induced aging.

Treatment	Model	Dose and administration route	Treatment period	Main outcomes	Ref.
Administration of cannabis-based products via parenteral routes					
THC in vehicle- ethanol:cremophor:saline, 1:1:18	12- and 18-month-old male C57BL/6J mice	3 mg per kg bodyweight per day s.c implanted osmotic minipumps	28 days	THC improves learning abilities in old mice and impairs learning abilities in young mice Beneficial effects of THC in cognition are dependent on CB1R signaling Upregulated of gene expression for: transthyretin, involved in protection against Alzheimer`s disease, BDNF, Caspase-1 in old mice	Bilkei-Gorzo et al. (2017)
THC THC: CBD 1:1 CBD	12- and 18-month-old male C57BL6/J mice	1 mg/kg/day THC s.c implanted osmotic minipumps	28 days	THC treatment improved spatial learning in mice THC alone was more efficient in improving spatial learning than combination with CBD.	Nidadavolu et al. (2021)
THC in vehicle- ethanol:cremophor:saline, 1:1:18	18-month-old Tg(Thy1-EGFP) MJrs/J (GFP-M) male mice	3 mg per kg bodyweight per day s.c implanted osmotic minipumps	28 days	Upreglation of BDNF levels Increased spine density and memory performance	Komorowska-Müller et al. (2023)
THC in vehicle- ethanol:cremophor:saline, 1:1:18	24-month-old female mice	0.002 mg/kg THC i.p. injected	One administration	Reversible volume changes and higher tissue density in 3 brain regions (the entorhinal cortex, the PFC and the posterior hippocampus) THC was neuroprotective	Sarne et al. (2018)
THC and vehicle (ethanol, saline)	19 months-old transgenic APP/PS1 mice (male and female)	0.002 or 0.02 mg/kg Intranasally	3 months once a day	Aβ1–40 and 1–42 peptides decreased THC treatment slowed the memory decline	Fihurka et al. (2022)
Cannabis plant material with Δ9-THC (10,3% THC and 0.05% CBD) Cannabis plant material with CBD (10,4% CBD and 0.36% THC)	19–20 months old, C57BL/J6 mice (male and female)	Vaporized Cannabis aerosols 25–220 ng/mL THC vs. CBD (plasma levels)	28 days	Cannabis plant with high dose of THC Antinociceptive effects A decrease in midbrain volume of the dopaminergic system and a reduction in gray matter volume Cannabis plant with high dose of CBD enhanced global network connectivity	Sadaka et al. (2023)
Cannabis cigarettes- 5.6% THC, 0% CBD, and 0.4% CBN.	24–28-month-old (aged) Fischer 344 x Brown Norway F1 hybrid rats (male and female)	Exposed to smoke from 3 to 5 cigarettes	One exposure	Unlike the effect on aged females, THC smoked enhanced working memory in aged males	Zequeira et al. (2025)
Oral administration of Cannabis based products					
THC in gelatine form	24–28-month-old (aged) Fischer 344 x Brown Norway F1 hybrid rats (male and female)	1 mg/kg, oral chronic treatment	Chronic treatment (3–5 weeks)	THC exposure enhanced working memory in both sexes	Zequeira et al. (2025)
EU-GMP certified Cannabis plant suspended in CMC-Na (15.6% THC and <1% CBD)	19 months-old Sprague Dawley male rats	6.25 and 25 mg/kg body weight Oral gavage administration	6 weeks once a day, 5 days a week with 2 days off	A safety profile at administered doses Reduction of apoptosis related to caspase-3 protein release in hippocampus region Reduction of TNF-α, COX-2, CD-68, IL-1β expression highlights an anti-inflammatory profile at DG region of hippocampus	Tudorancea et al. (2025)
CBD powder with vehicle (grapeseed oil) in Nutella	14 months-old C57BL/6 mice (at the beginning of the experiment) and 19 months-old (when tasks started). 21 months-old (histological analysis). For all these experiments they used male and female mice	20 mg/kg body weight Oral administration	7 months	CBD improved spatial memory consolidation via Morris Water Maze test Decresed GFAP in hippocampus region	Mirza Agha et al. (2025)
Extract of hemseeds *Cannabis sativa L*	Aging rat model (3 months-old Sprague Dawley male rats) induced by D-galactose treatment	200 mg/kg and 400 mg/kg of body weight Intragastric administration	Once daily for 14 weeks	Both doses improved performance in Morris Water Maze test Extract (400 mg/kg) reduced tau phosphorylation at Ser396 Extract suppressed expression of PS1 related to aged brain Antioxidant effect by regulating the expression of SOD and MDA proteins in the hippocampus	Chen et al. (2017)

i.p, intraperitoneally; s.c., subcutaneous; BDNF, brain-derived neurotrophic factor; CBD, cannabidiol; CBN, cannabinol; CB1R, cannabinoid receptor 1; CMC-Na, sodium carboxymethylcellulose; COX-2, cyclooxygenase 2; DG, dentate gyrus; GFAP, glial fibrillary acidic protein; IL-1β, interleukin 1β; MDA, malondialdehyde; PS1, presenilin 1; PFC, prefrontal cortex; Ref., references; SOD, superoxide dismutase; THC,tetrahydrocannabinol; TNF-α, tumor necrosis factor α.

Route and frequency of administration further modulated outcomes. Chronic regimens, particularly continuous or repeated daily dosing, were consistently associated with cognitive and neuroprotective effects than single-dose paradigms. Inhalational and intranasal administration at very low THC doses (0.002–0.02 mg/kg) also produced measurable benefits, including reduced amyloid burden and slowed cognitive decline, indicating that enhanced bioavailability can shift the effective dose range downward (Fihurka et al., 2022; Sadaka et al., 2023).

Oral administration (via gavage) in preclinical models introduced greater variability, with beneficial effects observed across a wider dose range. Low oral THC doses (1–2.5 mg/kg/day) improved working memory in aged rodents, whereas higher oral doses of cannabis extracts or CBD (200–400 mg/kg) were required to achieve anti-inflammatory, antioxidant, and neuroprotective effects in the hippocampus (Chen et al., 2017; Mirza Agha et al., 2025; Zequeira et al., 2025). Taking together, the studies summarized in Table 2 suggest that cannabinoid-related outcomes in aging cannot be attributed to dose magnitude alone. Rather, the reported effects appear to vary in relation to the route of administration and the duration of treatment. Across the included preclinical models, sustained exposure to relatively low THC doses was frequently associated with improvements in cognitive performance and synaptic markers, whereas studies employing higher doses more often reported effects on neuroinflammatory or neurodegeneration-related parameters. These observations should be interpreted as descriptive trends emerging from heterogeneous experimental designs, rather than as definitive dose–response relationships. However, it is important to emphasize that the routes of administration (i.p, s.c and gavage) employed in these preclinical models are not used for cannabinoid administration in humans. This fact limits the direct translation of these observations to the human context.

Moreover, the potential relevance of cannabinoid combinations should not be overlooked. Preclinical evidence indicates that specific ratios of THC and CBD may produce effects that differ from those observed when each compound is administered alone. In an experimental model of autoimmune encephalomyelitis, combined administration of purified THC and CBD at 10 mg/kg body weight each, delivered intraperitoneally for 7 consecutive days, was associated with a reduction in neuroinflammation. This effect was accompanied by decreased central levels of pro-inflammatory cytokines, including IL-17A, IFN-γ, TNF-α, IL-6, and IL-1β, alongside increased levels of the anti-inflammatory mediators IL-10 and TGF-β. Notably, these immunomodulatory effects were reported to depend on the activation of both CB1R and CB2R (Al-Ghezi et al., 2019).Additionally, the combination of THC and CBD at high doses (20 mg THC and 640 mg CBD) may produce undesirable effects in humans, including pronounced cognitive and psychomotor impairment (Zamarripa et al., 2023). Similar observations have also been reported in preclinical studies (20 mg/kg CBD and 10 mg/kg body weight THC) (Kasten et al., 2019). In this context, interactions between THC and CBD have been shown to influence neurological pathways, suggesting that different cannabinoid combinations may modify the overall pharmacological profile. Such observations highlight that cannabinoid effects emerge from integrated pharmacodynamic interactions rather than from isolated receptor activation.

At the same time, these interactions occur within a pharmacokinetic framework characterized by substantial variability. Cannabinoids undergo extensive hepatic metabolism, which represents an additional source of variability. Following oral THC administration, only a small fraction (4%–12%) reaches systemic circulation (AlTfaili and Ginsburg, 2023). Both THC and CBD undergo extensive hepatic metabolism via cytochrome P450 enzymes. THC is primarily metabolized by CYP2C9 and CYP3A4 into the active metabolite 11-hydroxy-THC (with psychoactive activity) and 11-carboxy-THC an inactive metabolite, whereas CBD is mainly metabolized by CYP3A4 and CYP2C19 into 7-hydroxyl-CBD and further inactivated into 7-carboxy-CBD, an inactive metabolite. Both compounds subsequently undergo phase II conjugation and are predominantly excreted via the biliary route (Lucas et al., 2018; Yau et al., 2023). In the aging organism, hepatic metabolism is further altered by reductions in liver mass and hepatic blood flow. Consistent with this, higher circulating levels of the inactive metabolite 11- hydroxy -THC have been reported in young adult rats compared with aged animals after oral THC administration (Zequeira et al., 2025). In parallel, the high lipophilicity of cannabinoids promotes accumulation in adipose tissue, thereby affecting distribution and elimination kinetics. Food intake represents an additional modulator, as postprandial administration has been shown to increase plasma CBD concentrations in both humans and rodents (Millar et al., 2018).

Beyond pharmacokinetics, interactions between cannabinoids and enzymatic systems critically influence therapeutic outcomes. CBD is a well-established inhibitor of cytochrome P450 enzymes, including CYP1A1, CYP1A2, CYP1B1, CYP2A6, CYP2C9, and CYP3A4, as well as glucuronidation enzymes such as UGT1A7 and UGT2B7 (Ujváry and Hanuš, 2016). These interactions may increase the risk of toxicity, either from CBD itself or from co-administered drugs. Clinically, this has been highlighted using pharmaceutical CBD (Epidyolex®) in epilepsy, where dose escalation has resulted in a four- to five-fold increase in the active clobazam metabolite N-clobazam, required dose adjustments due to significant adverse effects (Karaźniewicz-łada et al., 2021).

Due to their high lipophilicity, THC and CBD readily cross BBB and accumulate in central nervous system tissue (Calapai et al., 2020). Into the brain, cannabinoid is largely driven by passive diffusion across the BBB, while interactions with efflux transporters, including P-glycoprotein, adenosine triphosphate-binding cassette (ABC) transporters or breast cancer resistance proteins (BCRP), may modulate their subsequent central distribution. THC is a substrate of P-glycoprotein, which may limit or modulate its brain accumulation and contribute to interindividual variability in central effects. In contrast, CBD does not appear to be a substrate for major ABC transporters such as P-glycoprotein or BCRP, suggesting that its brain penetration is less influenced by active efflux mechanisms (Brzozowska et al., 2016; Calapai et al., 2020). This distinction may have important implications for CNS drug delivery and potential resistance phenomena, particularly in pathological conditions where transporter expression is altered (Zhou et al., 2025). Given these considerations, it is essential to address the direct impact of cannabinoid exposure on BBB structure and function. Evidence in this area remains limited and is largely derived from non-aging models. A recent study reported that THC administered escalating doses, from 2.5 to 10 mg/kg body weight over a 15-day period, reduced the expression of junctional adhesion molecule-A, occludin, and claudin-5 in the prefrontal cortex of mice. These alterations suggest compromised tight junction integrity. Mechanistically, the observed effects appear to be mediated by CB1R activation within this region. However, the specific dose responsible for these changes remains unclear. It is possible that the cumulative or progressive dosing regimen contributed to the outcome, particularly given that a dose of 10 mg/kg has been associated with adverse neurological effects in other experimental settings (Mohammadpanah et al., 2023; Zhang et al., 2025). Direct evidence addressing how THC modulates BBB structure and function during aging remains scarce. Moreover, these limitations highlight the importance of considering the pharmacokinetic properties of cannabinoids, which critically shape dosing regimens across different neuropathological conditions related to aging. This aspect becomes especially relevant in aging, where physiological alterations in key organs involved in drug absorption, distribution, metabolism, and elimination may substantially influence cannabinoid bioavailability, tissue exposure, and ultimately their impact on BBB function. On the other hand, CBD appears to have beneficial effects in maintaining BBB integrity, as described above. However, the potential role of CBD in maintaining BBB integrity remains largely inferred from its anti-inflammatory, antioxidant, and neuroprotective properties, and warrants further investigation in targeted models, particularly in the context of aging.

Considering the above observations, the route of administration emerges as a critical determinant when aiming to achieve consistent neurological effects of cannabinoids in the aging population. Oral administration is associated with substantial and often unpredictable variability in response to THC and CBD, largely due to first-pass hepatic metabolism, age-related alterations in liver function, and the high potential for drug–drug interactions in polymedicated patients. In contrast, sublingual formulations, such as cannabinoid tinctures, and oromucosal preparations, may offer more predictable pharmacokinetic profiles by partially bypassing first-pass metabolism and enabling more direct systemic absorption (Kaufmann et al., 2022). Although inhalation remains the most common route of administration in the general population for recreational use, it is not an appropriate option for the elderly. Beyond the well-recognized respiratory and cardiovascular risks, specific physicochemical properties of cannabinoids—particularly THC—further limit the suitability of this route. The high lipophilicity and rapid pulmonary absorption of THC lead to abrupt increases in plasma and brain concentrations, resulting in peak-related central effects that may be difficult to control in older individuals (Wolfe et al., 2023). This is especially relevant when using herbal cannabis preparations or THC-rich extracts, where dose standardization is limited and exposure may be highly variable. Additionally, emerging formulation strategies based on nanotechnology have gained increasing attention to further optimize cannabinoid delivery. Lipid-based nanocarriers, nanoemulsions, and polymeric nanoparticles have been shown to enhance solubility, improve gastrointestinal absorption, and reduce interindividual pharmacokinetic variability. By stabilizing systemic exposure and enabling controlled or sustained release, these approaches may contribute to more consistent central nervous system effects, which is particularly relevant in the context of aging, where physiological variability is pronounced. Moreover, advanced platforms incorporating targeting ligands or stimuli-responsive properties offer the potential to refine tissue distribution and minimize off-target effects (Stanciu et al., 2026). Although these strategies remain largely at the preclinical or early translational stage, they represent a promising direction for improving the neurological efficacy and predictability of cannabinoid-based therapies.

### 4.3 Cognitive effects of Cannabis-based products

Although human studies have documented meaningful benefits in several conditions, particularly in pain management, these findings are often accompanied by reports of cognitive and behavioral adverse effects, which complicate the overall interpretation of clinical outcomes. A notable example comes from a systematic review including 47 randomized controlled trials in pain research, which identified “cognitive or attention disturbances” as among the most frequently reported adverse events (Stockings et al., 2018). Importantly, many of these adverse effects were derived from self-reported experiences of the participants, which introduces variability in both perception and reporting accuracy. Moreover, the review encompassed a wide range of cannabinoid formulations and combinations, including isolated compounds and complex phytochemical extracts. This heterogeneity makes it difficult to attribute specific adverse effects to a particular cannabinoid or to a defined dosing regimen. Additional insight comes from a meta-analysis of cannabinoid use in chronic pain, which documented cognitive adverse events including disorientation, memory impairment, and confusion. Notably, memory impairment showed the highest prevalence among these effects, with an estimated rate of 5.3%. The analysis also indicated that this adverse event occurred most frequently in users of herbal cannabis formulations. The authors acknowledged that many of the included studies employed non-comparative designs, a limitation that reduces confidence in causal inferences and contributes to the interpretative uncertainty surrounding these results (Zeraatkar et al., 2022).

Clinical studies evaluating adverse responses to chronic cannabinoid use have predominantly been conducted in patients with cancer-related or other forms of chronic pain, where the primary outcomes focus on analgesic efficacy rather than isolated cognitive effects. Moreover, chronic pain itself has been associated with impairments in attention, memory, and other cognitive domains, as documented in clinical literature on pain-related cognitive dysfunction, which may confound the interpretation of neurobehavioral outcomes in these patient populations (Patel et al., 2025). Considering these limitations, reliance on clinical data alone may not yield definitive insights into the neurocognitive safety profile of cannabinoid-based interventions. These meta-analyses, while highly informative for understanding safety signals in chronic pain populations, also tend to highlight cognitive adverse events more prominently than potential benefits. As a result, they may inadvertently shape a cautious perception of cannabinoid-based therapies. Such perceptions can influence clinical decision-making and may restrict the evaluation of these compounds in older adults, a group in which neurological disorders are increasingly common. This goal must be corrected, given that preclinical studies show neuroprotective potential in cannabinoids, but existing clinical studies do not provide sufficient and robust data to support meta-analyses with high decision-making power and solid conclusions, especially on neurodegenerative pathologies. On the other hand, there are several clinical studies that reported favorable outcomes with synthetic THC analogues. Notably, nabilone, approved by the Food and Drug Administration for refractory chemotherapy-induced nausea and vomiting, has shown potential benefits in improving neuromotor symptoms in Parkinson disease (Peball et al., 2020). Additionally, another synthetic form of THC has attracted growing interest for its potential to modulate symptoms associated with neurodegenerative disorders in both clinical and preclinical settings-dronabinol. In clinical studies involving Alzheimer disease, dronabinol has been associated with improvements in behavioral disturbances, including agitation and alterations in circadian activity, with generally good tolerability (Walther et al., 2006). In parallel, experimental models of Parkinson disease using dronabinol suggest that cannabinoid receptor activation may attenuate motor impairment and exert neuroprotective effects on dopaminergic neurons (Fagan and Campbell, 2014).

In summary, the current evidence on the cognitive effects of CBP remains heterogeneous and, at times, contradictory. This variability largely reflects differences in study design, patient populations, and cannabinoid formulations, limiting the ability to draw consistent conclusions.

### 4.4 Mental health disorders associated with Cannabis-based products use

Despite the fact that cannabis has been used by people across throughout history, high quality clinical studies using CBP have been limited as a consequence of multiple barriers: regulatory restrictions (Schedule I status in the US), the diversity of cannabinoid products, lack of standardization and limited funding (Abuhasira and Novack, 2025; Bridgeman and Abazia, 2017; Cooper et al., 2021; DeFilippis et al., 2020; Kulpa et al., 2026; Pressman et al., 2024). This situation has steadily improved across time. In December 2025, in the U.S, an executive order was signed to expedite its reclassification to a Schedule III drug, in order to facilitate its use in medical research (Presidential action - Executive order, 2025). Global overall consumption of medical cannabis-based products has steadily increased, particularly among the aging population (Kaskie et al., 2024; Pravosud et al., 2025; Yang et al., 2021). Across the United States, the prevalence of past-month cannabis use in the older population has increased, with significant growth observed across multiple demographic subgroups and chronic medical conditions, such as chronic pain, cancer, COPD, anxiety, posttraumatic stress disorder (PTSD), and sleep disorders (Han et al., 2025; Kaskie et al., 2024; Yang et al., 2021). More than half of older adults reporting medical cannabis use initiated use after the age of 60, the majority use Cannabis for medical reasons rather than recreational purposes, prefer CBD-only products, oral and topical formulations over inhaled forms (Han et al., 2025; Page et al., 2020; Yang et al., 2021). A 2024 Australian study highlighted a difference in the way participants preferred different routes of administration. Thus, younger, male participants opted for THC-dominant inhaled routes of administration, and older, female participants opted for CBD-dominant oral routes of administration (Trevitt et al., 2024).

However, as opposed to past users and never users, current Cannabis use in older adults is also associated with higher rates of mental health disorders (Pravosud et al., 2025; Tourjman et al., 2023; Wolfe et al., 2023). Recent meta-analyses and cohort studies also reinforce a strong association between Cannabis use and major depressive disorders, with evidence suggesting that self-medication is a significant driver of use among those with mood disorders and is associated with a worsened course and increase severity of symptoms. Causality remains difficult to establish due to confounding and bidirectional relationships (Choi et al., 2016; Churchill et al., 2025; Gorfinkel et al., 2020; Osborn et al., 2015; Pacek et al., 2013; Tourjman et al., 2023).

Across the lifespan, the use of CBP varies greatly. In therapeutic field, CBD-dominant preparations are more frequently utilized, especially among patients with cancer. Currently, clinically approved cannabinoid-based medications include purified cannabidiol (Epidiolex®/Epidyolex®), THC-containing formulations such as dronabinol (Marinol®, Syndros®), the synthetic analogue nabilone (Cesamet®), and the THC/CBD combination nabiximols (Sativex®) (Baldwin et al., 2024). Beyond these, various standardized extracts and medical cannabis preparations are used in certain jurisdictions, although their clinical evidence remains heterogeneous and indication specific. Among approved cannabinoid-based medications, purified cannabidiol (Epidiolex®/Epidyolex®) is currently the most widely used, largely due to its robust clinical evidence and regulatory approval for treatment-resistant epilepsies. The drug is generally well tolerated, although oral administration is associated with adverse effects such as somnolence, diarrhea, fatigue, drug interactions, and liver enzyme elevations, with limited detail on toxicity provided in the product information. Due to the alcohol content, a clear contraindication for this product is disulfiram or metronidazole administered concurrently, which is especially common among the adult population. Thus, in the pediatric population, pharmaceutical-grade CBD oral solutions are used for drug-resistant epilepsy associated with Lennox-Gastaut syndrome, Dravet syndrome, and tuberous sclerosis complex. Off-label use in this population is reported, such as autism spectrum disorders, cancer-related symptoms, and cerebral palsy, but the safety data is limited (Approved Drug Products with Therapeutic Equivalence Evaluations | Orange Book, n.d.; Chhabra et al., 2024; Hsu et al., 2025). Converging evidence from human and animal models indicates that BBB dysfunction is present in at least a subset of individuals with these conditions (Aragón-González et al., 2022; Cafri et al., 2025; Curatolo et al., 2022; DiStasio et al., 2019; Eshraghi et al., 2020; Fiorentino et al., 2016; Gozal et al., 2021; Hsu et al., 2025; Kealy et al., 2020; Löscher and Friedman, 2020; Memis et al., 2022; Schoknecht and Shalev, 2012; Swissa et al., 2019). Young adults show higher rates of recreational and medical *Cannabis* use, with a preference for inhaled products and edibles, as we above mentioned. Common medical symptoms addressed include anxiety, pain and sleep disorders. Globally, cannabis flower remains the predominant form of cannabinoid-based product use, both in European countries and throughout the Americas, including Canada. While smoking remains the most common route of administration for recreational Cannabis use, medical use is predominantly based on oral formulations, particularly tinctures and oil extracts (Baldwin et al., 2024; Europe Medical Cannabis Market Size, Share & Analysis, 2033). Early initiation, increased THC concentration and higher potency of use are associated with an increased risk of problematic use and cannabis use disorder (Haug et al., 2017; Livne et al., 2026; Ueno et al., 2021). In the middle-aged population, there is a shift toward medical use of Cannabis, mainly for chronic pain, insomnia, anxiety, and cancer-related symptoms. Also, this group is likely to receive healthcare provider recommendations (Livne et al., 2026).

Both the pediatric and geriatric populations demonstrate heightened vulnerability to THC-related adverse effects, creating a U-shaped risk curve across the lifespan. In children and adolescents, THC exposure is associated with acute toxicity, long-lasting cognitive and psychiatric disturbances, and disruption of the neurodevelopmental processes, such as synaptic pruning and neurotransmitter signaling, though some changes could be reversible with cessation (Blohm et al., 2019; Hurd, 2025; Murray et al., 2022). In older adults, THC can increase the risk of neuropsychiatric symptoms, such as confusion, psychomimetic effects, along with an increased risk of falling, drug-drug interactions via CYP250 enzymes, altered pharmacodynamics, necessitating cautious dosing and monitoring, especially in those with frailty or comorbidities (Hsu et al., 2025; Page et al., 2020; Velayudhan et al., 2021b). Taking into consideration the numerous cannabinoid-based formulations, their complex effects on the human population across the lifespan, the difficulties of translating results from the preclinical to the clinical models, and the changing global attitudes toward cannabis and cannabis based medical products, we can observe why there is a paucity of high-quality clinical research of cannabinoids in old age populations (Minerbi et al., 2019; Pisani et al., 2021; Velayudhan et al., 2021a; Wolfe et al., 2023). Most clinical studies that tackle this subject are small, heterogenous, and often lack rigorous design, with few that fit the criteria of adequately powered randomized controlled trials and with limited follow-up. Many studies do not consistently address harms, have inadequate adjustment for confounding, include mixed-age populations, thus making it difficult to draw firm conclusions about efficacy and safety for cannabis-based products in the elderly population (Beauchet, 2018; Binkowska et al., 2025; Bosnjak Kuharic et al., 2021; Minerbi et al., 2019; Page et al., 2020; Pisani et al., 2021; van den Elsen et al., 2014; Velayudhan et al., 2021a; Wolfe et al., 2023).

Preclinical models frequently demonstrate potentially promising effects of cannabinoids, but these findings are not reliably translated in clinical trials involving older adults (Gruber et al., 2016). Many of these difficulties are due to the limitations of the animal models to accurately capture the physiological and pharmacokinetic differences, the heterogeneity of clinical populations, and the safety and adverse event profiles (sedation, falls and cognitive impairment may be under-recognized or less severe in animal models) (Beauchet, 2018; Binkowska et al., 2025; Hsu et al., 2025; Page et al., 2020; Pisani et al., 2021; Pravosud et al., 2025; Velayudhan et al., 2021a; Wolfe et al., 2023).

Additionally, animal studies often use purified cannabinoids at controlled doses, whereas clinical use involves variable formulations, routes and dosages, with oral bioavailability and pharmacodynamics differing significantly in the older adult population (Binkowska et al., 2025; Pocuca et al., 2021; Velayudhan et al., 2021a).

Although the only recommended use of a cannabis-based product in the elderly population is the use of prescription CBD for seizure management in Lennox-Gastaut syndrome, Dravet syndrome, and tuberous sclerosis complex, there are numerous off-label uses that show potential benefits: chronic pain, appetite stimulation, behavioral symptoms of dementia, anxiety and sleep disoders (Hsu et al., 2025; Livne et al., 2026; Minerbi et al., 2019; Page et al., 2020; Tumati et al., 2022; Velayudhan et al., 2021a; Wolfe et al., 2023; Yang et al., 2021). According the American Heart Association, while there is interest in the potential benefits of CBP for the management of neuropathic pain and age-related diseases, these uses lack sufficient regulatory approval and robust evidence in the elderly (Page et al., 2020).

Overall, the benefit-to-risk ratio of cannabinoids in the elderly remains unclear, partly due to these research gaps, and well-designed, age-specific clinical trials are needed to inform clinical practice (Beauchet, 2018; Binkowska et al., 2025; Bosnjak Kuharic et al., 2021; Minerbi et al., 2019; Page et al., 2020; Pisani et al., 2021; van den Elsen et al., 2014; Velayudhan et al., 2021a; Wolfe et al., 2023).

## 5 Conclusion

Overall, the evidence reviewed here indicates that cannabinoids exert distinct and dose-dependent effects on the aging brain, with THC and CBD displaying divergent neurological profiles. For THC, preclinical studies consistently support a biphasic response. Moderate to high doses (≥10 mg/kg body weight), particularly after intraperitoneal or subcutaneous administration, are associated with less favorable outcomes, including neuroinflammatory signaling, glial activation, and functional deficits. In contrast, low-dose THC exposure, typically within the 0.002–3 mg/kg body weight range, appears to engage adaptive and potentially neuroprotective mechanisms in aged models. These observations support the existence of a narrow, context-dependent therapeutic window.

By comparison, CBD demonstrates a more consistently favorable neurological profile in preclinical aging models. Its effects include attenuation of neuroinflammation, reduction of oxidative stress, modulation of glial reactivity, and improvement of cognitive performance. These actions occur largely independently of CB1R activation and involve alternative pathways, including indirect endocannabinoid modulation, PPARγ signaling, and redox-sensitive mechanisms.

Importantly, the translational potential of cannabinoids is constrained by several pharmacological challenges. Notably, oral administration by gavage appears to represent a safer experimental approach, as it has not been associated with overt negative neurocognitive effects in preclinical aging models. In addition, outcomes obtained following oral administration are inherently more relevant for clinical translation, as this route better reflects human exposure patterns. In contrast, intraperitoneal or subcutaneous administration, although widely used in experimental settings, is not employed in clinical practice and may exaggerate central exposure and adverse effects due to distinct pharmacokinetic profiles. These considerations further underscore the importance of administration route as a critical variable influencing both the interpretation of preclinical findings and their translational relevance to human cannabinoid use. Moreover, the complex and age-sensitive pharmacokinetics, variability in bioavailability across routes of administration, and alterations in drug metabolism and distribution associated with aging. Additionally, changes in BBB permeability and organ function may differentially affect cerebral exposure to THC and CBD. Together, these considerations complicate dose extrapolation from preclinical models to clinical settings and underscore the need for age-adapted, cannabinoid-specific dosing strategies to support safe and effective translation.

## Glossary

2-AG: 2-arachidonoylglycerol
5-HT1A: 5-hydroxytryptamine receptor 1A (serotonin receptor 1A)
AEA: anandamide
BBB: blood–brain barrier
BDNF: brain-derived neurotrophic factor
BrdU: bromodeoxyuridine
CB1R: cannabinoid receptor 1
CB2R: cannabinoid receptor 2
CBD: cannabidiol
CBP: cannabis-based products
CNS: central nervous system
COX-2: cyclooxygenase-2
DCX: doublecortin
ECS: endocannabinoid system
FAAH: fatty acid amide hydrolase
GFAP: glial fibrillary acidic protein
GSH/GSSG: reduced glutathione/oxidized glutathione ratio
GSK-3β: glycogen synthase kinase 3 beta
IFG: inflammaging
IGF-2: insulin-like growth factor 2
IL-1β: interleukin-1β
IL-6: interleukin-6
LPS: lipopolysaccharide
MDMA: 3,4-methylenedioxymethamphetamine
MWT: Morris water maze (test)
NF-κB: nuclear factor kappa-light-chain-enhancer of activated B cells
NO: nitric oxide
NOR: novel object recognition (test)
Ph. Eur.: European Pharmacopoeia
PPARγ: peroxisome proliferator-activated receptor gamma
ROS: reactive oxygen species
THC: tetrahydrocannabinol
TNF-α: tumor necrosis factor-α
Ttr: transthyretin
TuJ-1: neuron-specific class III beta-tubulin
VCAM-1: vascular cell adhesion molecule-1
